# Three days of intermittent stretching after muscle disuse alters the proteins involved in force transmission in muscle fibers in weanling rats

**DOI:** 10.1590/1414-431X20154118

**Published:** 2015-12-04

**Authors:** M.C.S. Gianelo, J.C. Polizzelo, D. Chesca, A.C. Mattiello-Sverzut

**Affiliations:** 1 Programa de Pós-Graduação em Reabilitação e Desempenho Funcional, Faculdade de Medicina de Ribeirão Preto, Universidade de São Paulo, Ribeirão Preto, SP, Brasil; 2 Departamento de Patologia, Faculdade de Medicina de Ribeirão Preto, Universidade de São Paulo, Ribeirão Preto, SP, Brasil; 3 Departamento de Biomecânica, Medicina e Reabilitação do Aparelho Locomotor, Faculdade de Medicina de Ribeirão Preto, Universidade de São Paulo, Ribeirão Preto, SP, Brasil

**Keywords:** Immobilization, Vimentin, Desmin, Intermittent stretching, Collagen

## Abstract

The aim of this study was to determine the effects of intermittent passive manual stretching on various proteins involved in force transmission in skeletal muscle. Female Wistar weanling rats were randomly assigned to 5 groups: 2 control groups containing 21- and 30-day-old rats that received neither immobilization nor stretching, and 3 test groups that received 1) passive stretching over 3 days, 2) immobilization for 7 days and then passive stretching over 3 days, or 3) immobilization for 7 days. Maximal plantar flexion in the right hind limb was imposed, and the stretching protocol of 10 repetitions of 30 s stretches was applied. The soleus muscles were harvested and processed for HE and picrosirius staining; immunohistochemical analysis of collagen types I, III, IV, desmin, and vimentin; and immunofluorescence labeling of dystrophin and CD68. The numbers of desmin- and vimentin-positive cells were significantly decreased compared with those in the control following immobilization, regardless of whether stretching was applied (P<0.05). In addition, the semi-quantitative analysis showed that collagen type I was increased and type IV was decreased in the immobilized animals, regardless of whether the stretching protocol was applied. In conclusion, the largest changes in response to stretching were observed in muscles that had been previously immobilized, and the stretching protocol applied here did not mitigate the immobilization-induced muscle changes. Muscle disuse adversely affected several proteins involved in the transmission of forces between the intracellular and extracellular compartments. Thus, the 3-day rehabilitation period tested here did not provide sufficient time for the muscles to recover from the disuse maladaptations in animals undergoing postnatal development.

## Introduction

During the treatment of skeletal muscle diseases, intermittent stretching is frequently used as part of the physical rehabilitation for infants and adults. The extent of muscle damage that occurs during these diseases can be characterized by the degree of involvement of the different muscle components, such as the membrane, sarcomere, and extracellular matrix. These components and their constituents have been extensively studied in humans and adult animals (1-5). The adaptive mechanisms involved in muscle recovery have also been extensively examined (6-8). During myogenesis, the number, size, and metabolic phenotype of muscle fibers (9) are defined at distinct stages of fetal development. After myogenesis, during the postnatal period, the muscles respond to hypertrophy by incorporating myonuclei from satellite cells (10). Furthermore, muscle fiber innervation may be modified in response to environmental stimuli, including stimuli from disease and pathological damage (11). Therefore, any pathological tissue adaptation that occurs during the postnatal period may impair the development of motor skills.

Our group has published several studies on muscle plasticity induced by distinct training protocols in both newly weaned (12,13) and adult (14,15) rats. Benedini-Elias et al. (12) found greater hypertrophy in response to eccentric exercise than to passive stretching in weanling animals whose hind limbs had been immobilized for 10 days before beginning rehabilitation. However, a similar approach used on adult animals produced different results (15). One recent study showed that continuous stretching applied to weanling animals post-immobilization reduced the hind limb support on the ground (13). Moreover, an increased number of macrophages was observed in those animals. These results indicated that the specific type and intensity of movements in the rehabilitation protocol adversely affected the gait function in weanling rats.

In the aforementioned studies, the immunohistochemical analysis showed no differences in the collagen I to collagen III expression ratios in the soleus muscle, regardless of the type of rehabilitative treatment applied, both in weanling and adult animals (13,14). Collagen mediates the initial passive transmission of force, which is then transmitted by membrane proteins to the cytoskeletal proteins (16). Therefore, in the present study, we examined the levels of costamere proteins, extracellular matrix components, and intermediate filaments in order to determine 1) the morphological effects of intermittent passive manual stretching applied over 3 days on the soleus muscles of naive weanling rats, and 2) the extent of soleus muscle damage induced by intermittent passive manual stretching applied over 3 days in weanling rats whose hind limbs had been first immobilized in plantar flexion.

## Material and Methods

### Animals and study groups

Twenty 21-day-old albino Wistar rats (*Rattus norvegicus*) immediately after weaning on day 21 were provided by the Central Animal Campus Hall of Ribeirão Preto-USP and used in this study. The rats were housed in pairs in 41×34×16-cm cages and provided free access to pelleted food and water. All appropriate procedures for cleaning and adaptation of the animals to the cages were followed. The protocol for this study was approved by the Ethics Committee on Animal Experimentation (CETEA; #043/2007). The animals were randomly divided into 5 groups: an initial control group (CG21; 21-day-old rats), an immobilized group (IG; 21-day-old rats that were immobilized for 7 days), an immobilized and stretched group (ISG; 21-day-old rats that were immobilized for 7 days and rehabilitated by stretching for 3 days), a stretched group (SG; 21-day-old rats that were not immobilized for 7 days and were stretched for 3 days), and a final control group (CG30; 30-day-old rats). The animals in the final group (CG30) progressed through the normal growth and development of weanling rats, and this group was mainly used to obtain control values for the intermediate filaments. After the intervention period, all of the animals were euthanized by administering an intraperitoneal overdose of a ketamine and xylazine mixture.

### Immobilization technique

After induction of anesthesia with an intraperitoneal dose of ketamine and xylazine (0.1 mL/10 g), the right hind limb of each animal was immobilized at maximal plantar flexion for 7 days, as previously described (17). The immobilization apparatus consisted of two parts: a top part that was similar to a cotton shirt and allowed the animal to freely move its head and upper limbs, and a lower part divided into anterior and posterior regions that consisted of a steel mesh with taped edges to prevent injuries to the animals. In addition, a “cotton cushion” was placed in front to protect the anterior compartment of the limb. The animals were kept immobilized for 7 consecutive days. The immobilization set-up did not prevent the animals from feeding or moving within the cage.

### Manual passive stretching

A manual force (not quantified) was applied to generate dorsiflexion until full knee extension. This procedure generated passive stretching of the triceps sural muscle. The stretching program, which was implemented in the morning, consisted of a daily series of 10 repetitions for 30 s each at 30-s intervals over 3 consecutive days.

### Soleus muscle collection

On the third day of rehabilitation, immediately after the stretching session, the animals were euthanized. The soleus muscles were removed, and fragments of the muscles were immersed in talc and frozen in liquid nitrogen. The muscle fragments were stored at -80°C until further processing.

### Histochemical and immunohistochemical staining

The muscle samples were sectioned in a cryostat at -25°C (Leica CM 1850 UV, Leica Microsystems, Germany) and 5-µm-thick sections were collected on 26×76-mm slides. For the histochemical analysis, sections were stained with hematoxylin-eosin (HE) and picrosirius red (18). For the immunohistochemical analysis, sections were stained with antibodies against collagen types I, III, and IV, desmin, and vimentin. These procedures were performed at the Laboratório de Neuropatologia, Departamento de Patologia, Faculdade de Medicina de Ribeirão Preto, Universidade de São Paulo, Brazil, following the standard protocols for skeletal muscle processing.

The slides were fixed in cold acetone for 10 min, washed with phosphate-buffered saline (PBS), and incubated in 3% H_2_O_2_ for 15 min to block endogenous peroxidase activity. Next, the slides were washed again and then blocked with normal horse serum (Vectastain ABC Kit, USA) for 60 min to prevent nonspecific binding. Excess liquid was removed, and different sections from each group were incubated with the following primary antibodies at 4°C overnight: monoclonal mouse anti-rat collagen type I (Sigma, USA; 1:18,000 dilution), type III (Sigma; 1:36,000 dilution), and type IV (Sigma; 1:800 dilution); mouse anti-human desmin (Dako, Denmark; 1:100 dilution); and mouse anti-human vimentin (Dako; 1:100 dilution). In the negative control (blank) sections, no primary antibody was added. Next, the sections were washed three times with Tris-buffered saline containing Tween 20 (TBST) and then incubated with secondary antibodies (Vectastain ABC kit) for 30 min, immediately washed three times, and incubated for 30 min with avidin and biotin (Vectastain ABC kit). Afterwards, the sections were washed with TBST and Tris-HCl and incubated with the chromogen diaminobenzidine for 5 min. Finally, the sections were counterstained with hematoxylin for 10 s, dehydrated and diaphanized, and mounted using Permount™ medium (Fisher Scientific, USA).

To assess the immunofluorescence, the muscle sections were stained with rabbit polyclonal anti-dystrophin antibody (Abcam, UK; 1:100 dilution) and mouse monoclonal anti-CD68 antibody (Abcam; 1:200). First, the sections were washed in TBS, fixed in TissueTek¯ Xpress¯ molecular fixative for 4 min, and washed twice with TBS. Next, the excess liquid was removed and the sections were incubated with the primary antibodies at 37°C for 2 h, washed three times with PBS for 5 min each, and then incubated for 45 min with the secondary antibodies (Alexa 488 goat anti-rabbit and Alexa 568 goat anti-mouse antibodies; Molecular Probes, USA). Finally, the sections were washed twice with TBS and mounted in Prolong¯ Gold Antifade reagent (Invitrogen, USA).

### Morphological and morphometric analyses

The muscle cross-sections were qualitatively and quantitatively analyzed under a light microscope (Leica DM 2500; Leica Microsystems) with the Leica LAS V3.8 software. The analyses were performed on three random fields of the soleus muscle obtained from the animals in each group. The general morphological aspects of the muscle tissue were evaluated mainly on the HE and picrosirius stained sections. The semi-quantitative grading of the collagen staining (types I, III, and IV) was performed by three independent observers. The results were classified as absent (-), weakly positive (±), slightly positive (+), moderately positive (++), or strongly positive (+++), following the classification described by Kurose et al. (19). Morphological changes in the muscle cells, such as the presence of inclusion bodies, fragmentation, and central core lesions, were quantified by a single observer who examined three random HE-stained fields of equal magnification. The mean number of cells positive for intermediate filaments (desmin or vimentin) was determined as the automatic values calculated by the Leica LAS V3.8 software for the positive labeling of cells with desmin or vimentin antibodies based on three analyzed fields for each animal. The macrophage density was calculated by manually counting all of the CD68-positive cells, again based on three analyzed fields for each animal. The dystrophin labeling defined the borders of the cell membrane. Statistical analyses were performed using R Core Team (2014) (R: A Language and Environment for Statistical Computing. R Foundation for Statistical Computing, Austria; http://www.R-project.org/). Kruskal-Wallis tests were used to compare nonparametric data from two or more groups. Dunn's post-tests were used to compare specific groups. The level of significance was set at 5%, and the confidence interval at 95% (95%CI).

## Results

**Figure 1:**
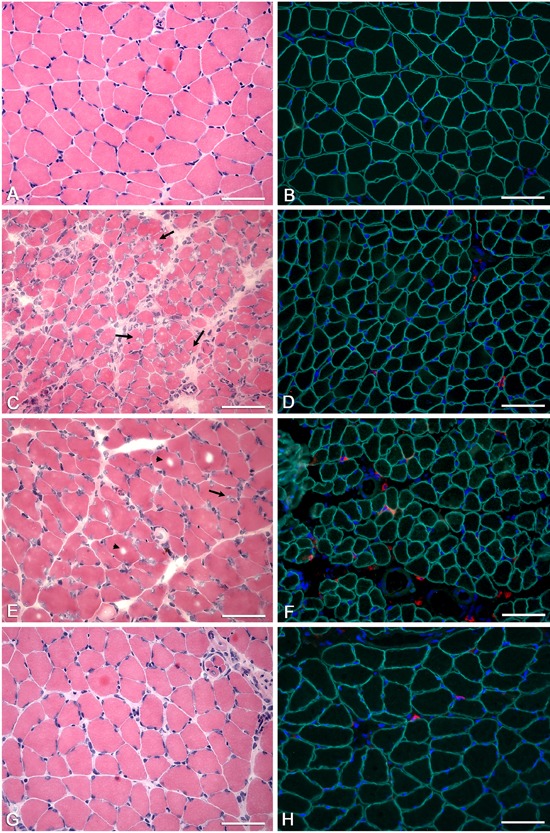
Photomicrographs of soleus muscle: hematoxylin-eosin (HE) staining and immunostaining for macrophages. Cross-sections of soleus muscle showing the general morphology (HE) and macrophage immunolabeling. *A*, *C*, *E*, *G*: HE staining; *B*, *D*, *F*, *H*: immunolabeling with rabbit anti-dystrophin (green) and mouse anti-CD68 (red) antibodies, and nuclear staining with DAPI (blue). *A* (CG30) and *B* (CG30) show polyhedral fibers and peripheral nuclei; *C* (IG) shows differences in fiber sizes, inclusion bodies (black arrow), central core lesion, and myonecrosis; *D* (IG) shows macrophage immunolabeling in the interstitial space; *E* (ISG) shows slight differences in fiber sizes, central cores (arrowhead), and inclusion bodies (black arrow); *F* (ISG) shows an increased number of macrophages; *G* (SG) and *H* (SG) show an absence of histopathological alterations. Bars: 50 µm. CG21: 21-day control group; CG30: 30-day control group; IG: immobilized group; ISG: immobilized and stretched group; SG: stretched group.

Histopathological changes, including inclusion bodies, cell fragmentation, and central core lesions, were observed in the muscles of the animals that were immobilized and then rehabilitated using stretching (Table 1 and Figure 1). The mean number of inclusion bodies was significantly larger in IG and ISG animals than in CG21 animals (IG×CG21 and ISG×CG21: P<0.05). Moreover, the ISG animals had a larger number of inclusion bodies than the CG30 animals (ISG×CG30: P<0.05). Cell fragmentation was also significantly higher in the muscles of the ISG animals than in the muscles of the control-group animals (ISG×CG21 and ISG×CG30: P<0.05). Similarly, the mean number of central core lesions was significantly larger in IG and ISG animals than in CG21, CG30 or SG animals (IG×CG21, IG×CG30, ISG×CG21, ISG×CG30, IG×SG, and ISG×SG: P<0.05). Macrophage density was also significantly different between the CG21 and CG30 animals and the ISG animals (ISG×CG21 and ISG×CG30: P<0.05; Table 1), as shown in Figure 1.

**Table:**
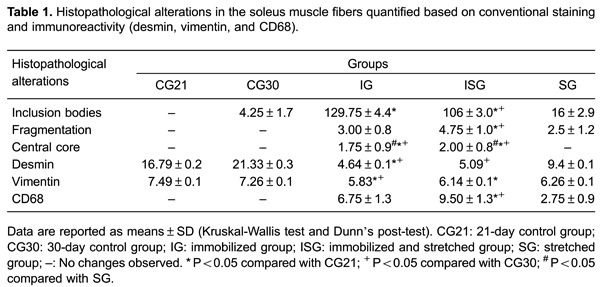

**Figure 2:**
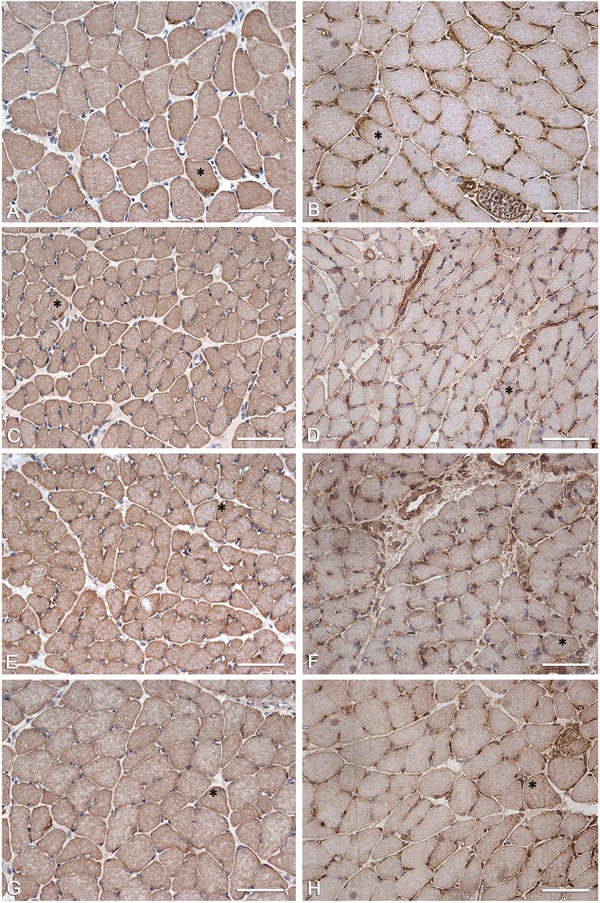
Photomicrographs of soleus muscle: immunostaining for desmin and vimentin. Cross-sections of soleus muscle were immunolabeled for intermediate filaments. *A*, *C*, *E*, *G*: desmin immunolabeling; *B*, *D*, *F*, *H*: vimentin immunolabeling. *A* (CG30), *B* (CG21), *C* (IG), *D* (IG), *E* (ISG), *F* (ISG), *G* (SG), and *H* (SG): desmin- and vimentin-positive cells are shown, and certain cells are more strongly stained than others (asterisks). Bars: 50 µm. CG21: 21-day control group; CG30: 30-day control group; IG: immobilized group; ISG: immobilized and stretched group; SG: stretched group.

The numbers of desmin-positive cells in the control animals were significantly different from those in IG animals, and those in the CG30 animals were different from ISG animals (CG21×IG, CG30×IG, and CG30×ISG: P<0.05; Table 1 and Figure 2). The numbers of vimentin-positive cells were also significantly different between the control groups and IG, as well as between CG21 and IG and ISG (CG21×IG, CG30×IG, and CG21×ISG: P<0.05; Table 1 and Figure 2).

**Figure 3:**
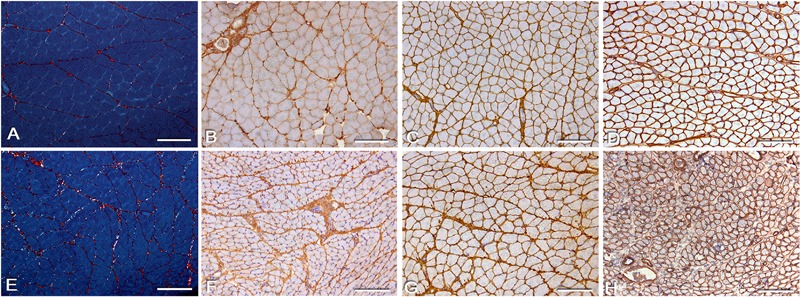
Photomicrographs of soleus muscle: labeling of connective tissue elements. Cross-sections of soleus muscle were stained with picrosirius red and immunolabeled for collagen types I, III, and IV. *A* and *E*: picrosirius red; *B* and *F*: collagen type I immunolabeling; *C* and *G*: collagen type III immunolabeling; *D* and *H*: collagen type IV immunolabeling. *A* (CG30) shows moderate to strong picrosirius red staining; *E* (ISG) shows strong picrosirius red staining; *B* (CG30) shows weak labeling for collagen type I; *F* (IG) shows moderate labeling for collagen type I; *C* (CG30) shows moderate labeling for collagen type III; *G* (SG) shows moderate to strong labeling for collagen type III; *D* (CG30) shows moderate to strong labeling for collagen type IV; *H* (ISG) shows moderate to strong labeling for collagen type IV. Bars: 50 µm. CG21: 21-day control group; CG30: 30-day control group; IG: immobilized group; ISG: immobilized and stretched group; SG: stretched group.

The semi-quantitative grading of the picrosirius red staining indicated that the fragments of soleus muscle from all groups showed moderate to strong positive staining (++/+++) (Table 2 and Figure 3). The staining for type I collagen was moderately positive (++) in IG and slightly positive (+) in all of the other groups (Table 2 and Figure 3). The staining for type III collagen was moderately positive (++) in all groups. The staining for type IV collagen was strongly positive (+++) in the CG21, CG30, and SG animals, while it was moderately positive (++) in IG and ISG animals (Table 2 and Figure 3).

**Table:**
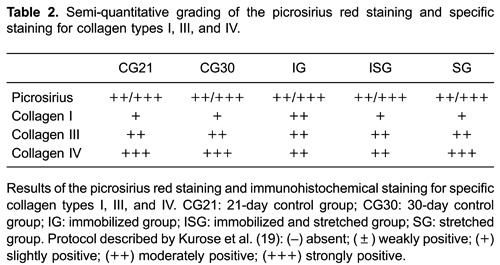

## Discussion

The results of this study indicate that the soleus muscle of newly weaned rats undergoes major morphological changes when subjected to therapeutic stretching after 7 days of disuse. In contrast, the cytoarchitecture of the muscle fibers in naive muscles (without disuse) was not affected by stretching. The major abnormalities found in the muscles of animals that received therapeutic stretching after immobilization were inclusion bodies, cells fragmentation, and central core lesions. These changes were also previously observed in adult (14,15) and newly weaned (12) animals. This suggests that micro-injuries occur in muscle fibers subjected to remobilization after immobilization, which was corroborated by the macrophage infiltration observed in those muscles. The increased susceptibility of the muscle fibers to degenerative cellular abnormalities may be associated with a reduction in type IV collagen, as observed in animals from the IG and ISG groups. However, this susceptibility could also be caused by an increase in the levels of type I collagen, as was found in the IG group. The transmission of forces between the intracellular and extracellular compartments appeared to be impaired in the muscles that were immobilized. The integrity of the costamere proteins may have been compromised, triggering cellular degeneration and necrosis (14).

Kannus et al. (20) showed that slow twitch muscles are particularly vulnerable to immobilization and that the fibers of these muscles are susceptible to architectural and histochemical alterations. According to Frimel et al. (21) and Mattiello-Sverzut et al. (22), hypokinesia can cause nuclear centralization in muscles. Kamiãska and Szyluk (23) reported that tenotomy-induced disuse of muscles inhibits or reduces their mechanical translation of stimuli. This may explain the increased expression of desmin and vimentin observed in the control groups in the present study. Disuse and/or reduced movement of muscle fibers has been reported to be associated with an increased expression of type I collagen, which could result in an increased resistance to movement (24). Thus, a vicious cycle is established: the accumulation of intramuscular connective tissue results in decreased blood flow and collapse of the capillary lumens, which contributes to an increase in the connective tissue synthesis (25). Therapeutic stretching after immobilization resulted in a decrease in the level of type IV collagen, but did not prevent muscle damage. Similar effects were also observed by Ahtikoski et al. (26) in adult animals whose hind limbs had been immobilized in a shortened position for 7 days, the same duration as was used here. The duration of rehabilitation was too short to complete the restructuring of the extracellular matrix elements.

In immature muscles, desmin and vimentin both exist in the Z line region. Vimentin is present in relatively larger amounts during embryogenesis, whereas desmin levels increase after muscle development and maturation (27,28). Stretching induces stimuli in both the longitudinal and transverse directions in muscle (29), thus mobilizing the sarcomeres, Z lines, and other intermediate filaments. Here, the levels of desmin and vimentin were lower in the muscles of animals subjected to immobilization than in the muscles of the control-group animals (21- and 30-day-old rats), indicating a counterbalancing effect of disuse. The expression levels of these proteins were not changed in the animals treated exclusively with stretching. In animals subjected to immobilization and stretching, the desmin expression did not increase markedly or reach the levels observed in the 30-day control group. These data suggest that a longer period (>3 days) of rehabilitation is needed to restore desmin levels and repair the architecture of the interstitial filaments, which would facilitate the mechanical transduction of signals between the intracellular and extracellular compartments. However, to determine the long-term effects of immobilization and stretching on the cytoarchitecture of the intermediate filaments, studies similar to the present one should be conducted over longer periods and incorporate additional biochemical and molecular biology analyses.

The results of the present study support the conclusion that intermittent passive manual stretching, when applied to healthy muscle, does not cause major morphological changes. However, when stretching is applied after 7 days of immobilization, it can cause micro-injuries and degenerative abnormalities in the costamere proteins, extracellular matrix, and intermediate filaments of muscle fibers. Remobilization for 3 days is not sufficient to restore the cytoarchitecture and integrity of the proteins involved in the transmission of forces between the intracellular and extracellular compartments. Therefore, a rehabilitation duration of >3 days may be necessary to achieve complete structural adaption in the muscles of animals during postnatal development.
